# Successful Retrieval of a Drifting Dislodged Leadless Pacemaker From the Left Common Iliac Artery

**DOI:** 10.7759/cureus.102444

**Published:** 2026-01-27

**Authors:** Michael W Alchaer, Christina Mayberry, Mackenzie Elting, Paul Farag, Thomas A Abbruzzese

**Affiliations:** 1 Department of General Surgery, HCA Healthcare/University of South Florida (USF) Morsani College of Medicine Graduate Medical Education (GME) HCA Florida Brandon Hospital, Brandon, USA; 2 Department of Medicine, DeBusk College of Osteopathic Medicine, Harrogate, USA; 3 Department of Medicine, College of Osteopathic Medicine, Nova Southeastern University, Fort Lauderdale, USA

**Keywords:** general and vascular surgery, iliac artery, leadless pacemaker implantation, pacemaker lead migration, surgical case reports

## Abstract

Leadless pacemakers (LPs) have become increasingly popular alternatives to conventional transvenous systems because they eliminate leads and generator pockets, reducing the risks of infection, lead fracture, and venous thrombosis. Large multicenter studies have shown lower overall complication rates and fewer reinterventions compared to traditional pacemakers. Despite these advantages, LPs are not free from risk. Migration or embolization of the device is an exceptionally rare but potentially life-threatening complication that requires prompt recognition and intervention. Most reported migrations involve the pulmonary vasculature; arterial migration is exceedingly uncommon.

We report the case of a 79-year-old woman with a history of atrial fibrillation and sinus pauses who was found to have imaging evidence of a foreign body in the left common iliac artery. Seven weeks earlier, she had undergone LP implantation. She was asymptomatic and hemodynamically stable on presentation. Computed tomography of the abdomen and pelvis revealed a 3-cm metallic density in the distal left common iliac artery. The patient underwent open retroperitoneal exploration with retrieval of the migrated pacemaker and removal of an associated thrombus, followed by primary arterial repair. Her postoperative course was uneventful, and she was discharged home on postoperative day 4 with intact distal perfusion.

Migration of an LP into the arterial circulation is exceptionally rare, with only isolated cases reported in the literature. Early identification and timely surgical intervention are essential to prevent ischemic complications.

This case underscores the importance of maintaining vigilance after device implantation and demonstrates that open surgical retrieval with primary arterial repair can be performed safely and effectively. Multidisciplinary collaboration between cardiology, vascular surgery, and radiology is crucial in managing these rare complications.

## Introduction

Leadless pacemakers (LPs) have emerged as a major advancement in cardiac rhythm management, offering a viable alternative to traditional transvenous systems. By eliminating the need for subcutaneous pockets and intravascular leads, LPs reduce the risk of infection, lead fracture, and venous thrombosis [1-3]. Large multicenter trials and real-world studies have demonstrated reductions of up to 48% in overall complications and significantly fewer revision procedures compared with conventional systems [4-6].

Despite these advantages, LP implantation is not without risk. The procedure requires a large-bore femoral venous sheath, predisposing patients to vascular injury in approximately 1%-2% of cases [7]. Device dislodgment or embolization occurs in less than 0.5% of patients, with most events involving migration to the pulmonary vasculature [8]. Such complications are typically managed by percutaneous snare retrieval under fluoroscopic guidance [9].

Reports of arterial migration of LPs are exceedingly rare, with only a few isolated cases described in the literature [10,11]. When migration occurs into the arterial system, it carries the potential for thrombosis, distal embolization, and limb ischemia, often requiring urgent surgical management [12,13]. The following case describes arterial migration of an LP to the left common iliac artery and highlights successful open surgical retrieval with primary arterial repair. This report contributes to the limited body of literature describing this rare and potentially life-threatening complication.

## Case presentation

A 79-year-old woman with a medical history significant for atrial fibrillation, sinus pauses, hypertension, diabetes mellitus, and multiple prior abdominal surgeries presented at the request of her cardiologist for evaluation of a suspected foreign body within the left common iliac artery. She had undergone LP implantation at another institution seven weeks earlier. Her past surgical history included total abdominal hysterectomy, cholecystectomy, appendectomy, right nephrectomy, thyroidectomy, and pacemaker placement.

On admission, she was asymptomatic. She denied fever, chills, abdominal pain, leg pain, numbness, weakness, syncope, or palpitations. Vital signs were normal. Cardiovascular examination showed an irregular rhythm with palpable dorsalis pedis and posterior tibial pulses bilaterally. Abdominal examination was benign. Laboratory studies were unremarkable.

**Figure 1:**
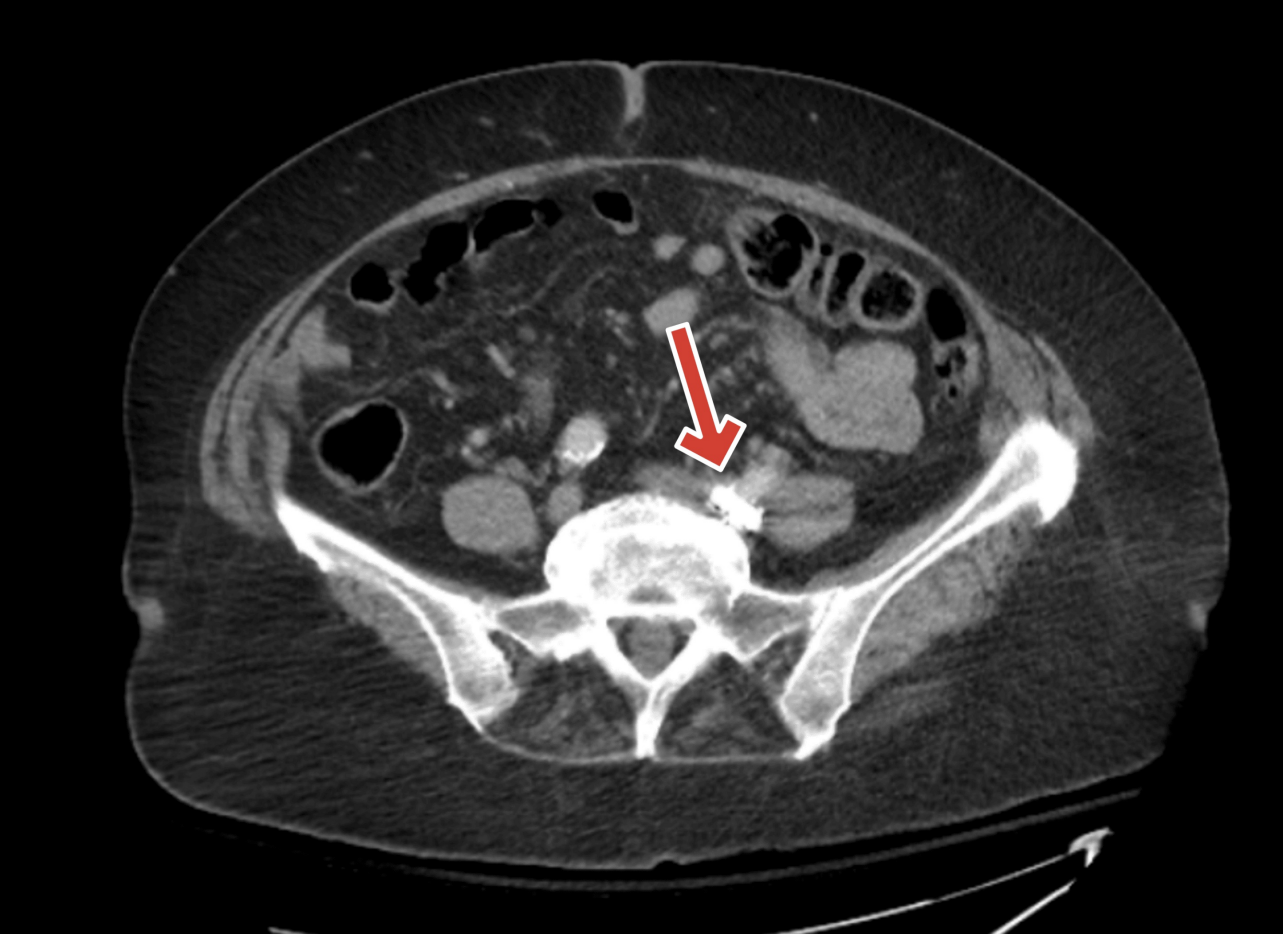
CT of the abdomen/pelvis with IV contrast with 3-cm radio-opaque density (arrow points at the foreign radio-opaque object) CT: computed tomography

Computed tomography of the abdomen and pelvis with IV contrast revealed a 3-cm metallic density in the distal left common iliac artery without evidence of bowel ischemia or acute intra-abdominal pathology (Figure 1). Incidental findings included sigmoid diverticulosis and an absent right kidney.

After a multidisciplinary discussion, the decision was made to proceed with the surgical removal of the migrated device.

Operative details

Under general anesthesia, a transverse lower abdominal incision was made. Dissection proceeded through the rectus muscle into the retroperitoneum. The peritoneum was mobilized, and the left common, internal, and external iliac arteries were carefully dissected and encircled with vessel loops. A Rummel tourniquet was placed distally on the common iliac artery, and proximal control was obtained.

**Figure 2:**
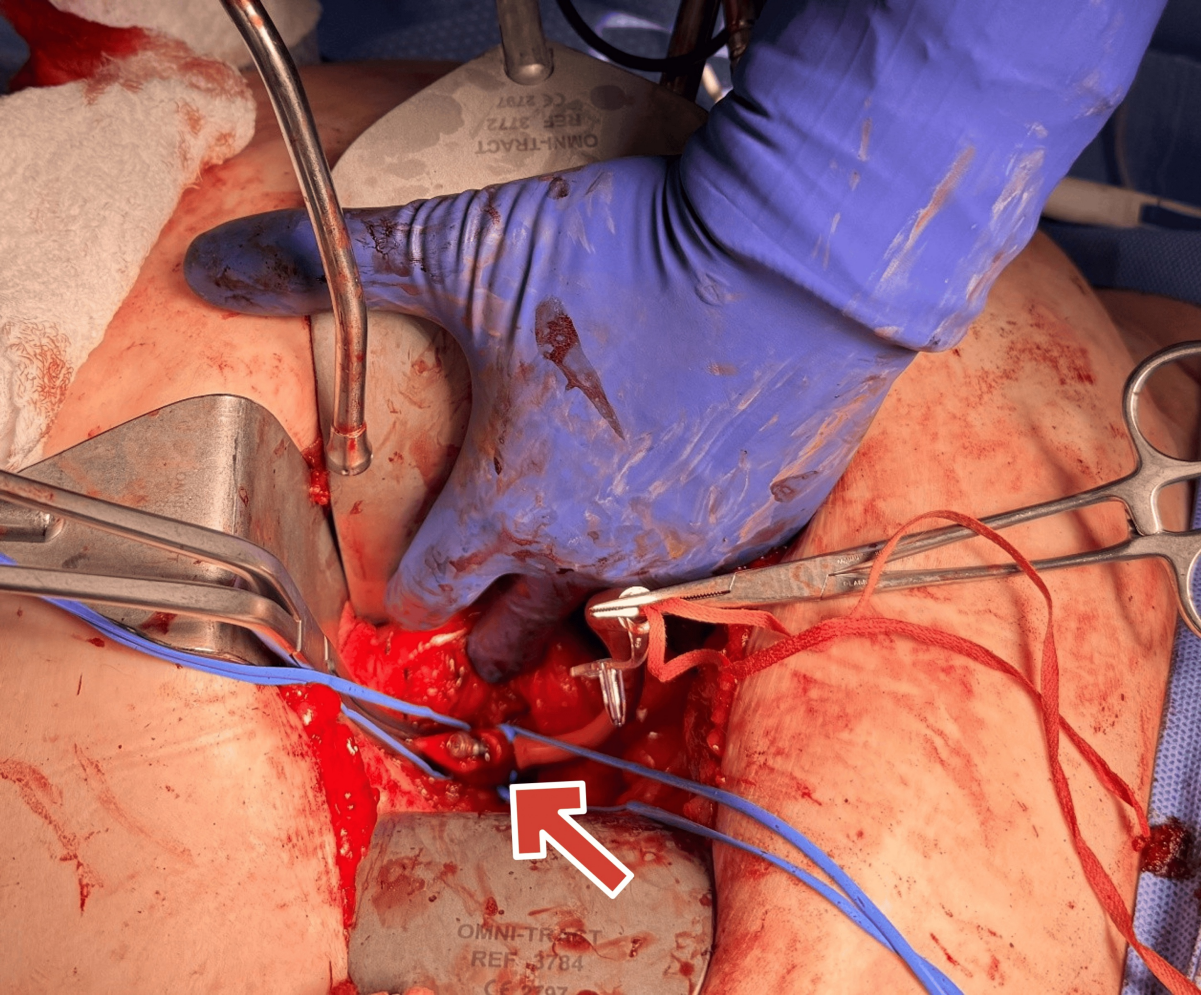
Intraoperative exposure showing leadless pacemaker lodged in the left external iliac artery (arrow points at the leadless pacemaker)

**Figure 3:**
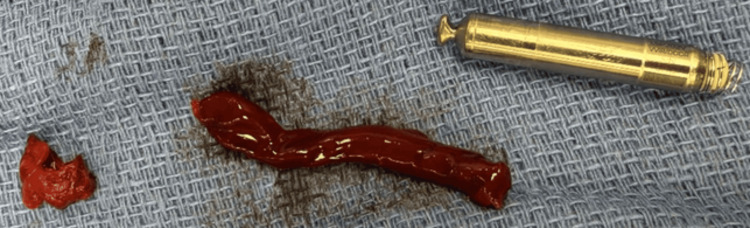
Removed leadless pacemaker with associated clot Left to right: two clots around the leadless pacemaker

Following systemic heparinization, an arteriotomy was made directly over the palpable device (Figure 2). The pacemaker and the associated clot covering it were removed (Figure 3). Back bleeding was brisk, and the artery was repaired with interrupted 4-0 Prolene sutures. Doppler confirmed excellent flow. Hemostasis was secured, the fascia was closed with a looped absorbable suture, and the skin was approximated with staples.

The postoperative course was completely benign, and the patient left home on postoperative day 4.

## Discussion

LPs provide clear advantages over traditional transvenous devices, largely by reducing lead- and pocket-related complications [6,14]. Nonetheless, complications related to vascular access remain an important concern. The use of a large-bore femoral sheath introduces a small but significant risk of vascular injury, reported in approximately 1%-2% of cases [2,7,9].

Device migration or embolization is an uncommon event, occurring in fewer than 0.5% of patients [8,10]. The majority of these migrations involve displacement into the pulmonary arterial system, where percutaneous retrieval using snares or endovascular techniques has been described with good success rates [9,11,15]. In contrast, migration into the arterial circulation is exceedingly rare and poses greater technical challenges. Only a handful of reports describe successful retrieval of LPs from the iliac or peripheral arteries [10,12].

In the present case, the pacemaker migrated into the left common iliac artery and was discovered incidentally in an asymptomatic patient. This underscores the importance of maintaining a high index of suspicion and utilizing imaging when patients present with unexplained vascular findings following device implantation [1,4]. Management strategy depends on device location, thrombus presence, and clinical stability. While percutaneous retrieval remains the preferred approach for venous or pulmonary embolization, open surgical removal provides a definitive solution in arterial cases, especially when accompanied by thrombus or risk of distal embolization [10,12].

This case highlights the importance of early recognition, multidisciplinary evaluation, and prompt intervention to prevent catastrophic complications. Collaboration among cardiology, vascular surgery, and radiology teams is crucial to ensure safe and effective management. As adoption of LPs continues to grow, awareness of this rare complication and strategies for retrieval are essential for improving outcomes.

## Conclusions

LPs represent an important advance in cardiac rhythm management, offering reduced complication rates compared to traditional systems. However, migration remains a rare but serious event. Arterial lodgment, particularly in the iliac system, is exceedingly rare and may not produce symptoms initially. Prompt recognition and surgical intervention are critical to preventing limb ischemia or systemic embolization. Our case demonstrates that open retroperitoneal retrieval with primary repair of the iliac artery can be performed safely and effectively. Clinicians should remain vigilant for this rare complication and engage a multidisciplinary team when managing such complex cases.
